# Comparison of FECPAK^G2^, a modified Mini-FLOTAC technique and combined sedimentation and flotation for the coproscopic examination of helminth eggs in horses

**DOI:** 10.1186/s13071-022-05266-y

**Published:** 2022-05-12

**Authors:** Heike Boelow, Jürgen Krücken, Eurion Thomas, Greg Mirams, Georg von Samson-Himmelstjerna

**Affiliations:** 1 Institute for Parasitology and Tropical Veterinary Medicine, Freie Universität Berlin, Robert-von-Ostertag-Str. 7–13, 14163 Berlin, Germany; 2 Techion UK, Peithyll Centre, Capel Dewi, Aberystwyth, SY23 3HU Wales UK; 3 Techion New Zealand, Invermay Agriculture Centre, Block A, 176 Puddle Alley, Mosgiel, 9092 New Zealand

**Keywords:** Strongylidae, *Parascaris *spp., Anoplocephalidae, FECPAK^G2^, Mini-FLOTAC, Combined sedimentation/flotation

## Abstract

**Background:**

Due to high prevalence of anthelmintic resistance in equine helminths, selective treatment is increasingly promoted and in some countries a positive infection diagnosis is mandatory before treatment. Selective treatment is typically recommended when the number of worm eggs per gram faeces (epg) exceeds a particular threshold. In the present study we compared the semi-quantitative sedimentation/flotation method with the quantitative methods Mini-FLOTAC and FECPAK^G2^ in terms of precision, sensitivity, inter-rater reliability and correlation of worm egg counts to improve the choice of optimal diagnostic tools.

**Methods:**

Using sedimentation/flotation (counting raw egg numbers up to 200), we investigated 1067 horse faecal samples using a modified Mini-FLOTAC approach (multiplication factor of 5 to calculate epgs from raw egg counts) and FECPAK^G2^ (multiplication factor of 45).

**Results:**

Five independent analyses of the same faecal sample with all three methods revealed that variance was highest for the sedimentation/flotation method while there were no significant differences between methods regarding the coefficient of variance. Sedimentation/flotation detected the highest number of samples positive for strongyle and *Parascaris* spp. eggs, followed by Mini-FLOTAC and FECPAK^G2^. Regarding Anoplocephalidae, no significant difference in frequency of positive samples was observed between Mini-FLOTAC and sedimentation/flotation. Cohen’s κ values comparing individual methods with the combined result of all three methods revealed almost perfect agreement (κ ≥ 0.94) for sedimentation/flotation and strong agreement for Mini-FLOTAC (κ ≥ 0.83) for strongyles and *Parascaris* spp. For FECPAK^G2^, moderate and weak agreements were found for the detection of strongyle (κ = 0.62) and *Parascaris* (κ = 0.51) eggs, respectively. Despite higher sensitivity, the Mini-FLOTAC mean epg was significantly lower than that with FECPAK^G2^ due to samples with > 200 raw egg counts by sedimentation/flotation, while in samples with lower egg shedding epgs were higher with Mini-FLOTAC than with FECPAK^G2^.

**Conclusions:**

For the simple detection of parasite eggs, for example, to treat foals infected with *Parascaris* spp., sedimentation/flotation is sufficient and more sensitive than the other two quantitative investigared in this study. Mini-FLOTAC is predicted to deliver more precise results in faecal egg count reduction tests due to higher raw egg counts. Finally, to identify animals with a strongyle epg above a certain threshold for treatment, FECPAK^G2^ delivered results comparable to Mini-FLOTAC.

**Grpahical Abstract:**

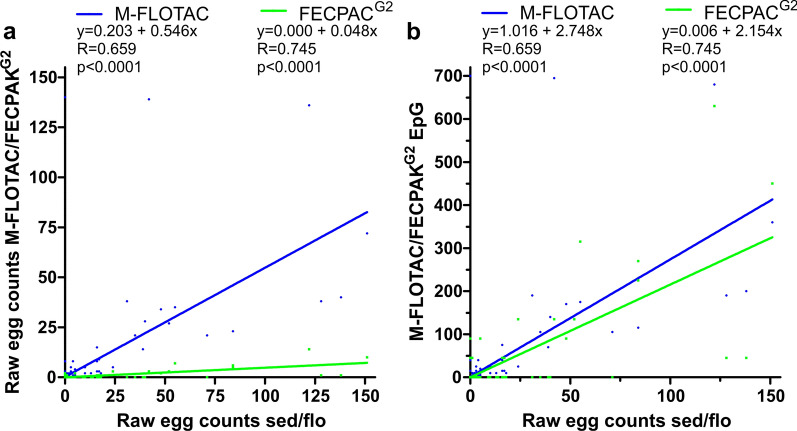

**Supplementary Information:**

The online version contains supplementary material available at 10.1186/s13071-022-05266-y.

## Background

The clinical symptoms caused or promoted by helminths in the horse include colic, constipation and diarrhoea [1, 2]. In some horses, there may also be non-specific symptoms, such as as reduced performance and emaciation, especially when infested with small strongyles [1, 3]. Meaningful worm control, therefore, requires reliable diagnostics. This is particularly necessary due to the increasingly encountered problem of anthelmintic resistance in parasitic nematodes [4–7]. New drugs, as alternatives to the established antiparasitics, as well as vaccines and new control measures are not available for equines or receive little research attention [7, 8]. Strategic deworming of horses, i.e. regular deworming at fixed times of the year without prior diagnosis, is still standard on many horse farms, which leads to the treatment of horses that have low or no detectable parasite burdens [9–12]. However, a targeted, rational use of medication in combination with appropriate management is indispensable for successful, efficient and sustainable deworming. Therefore, it is recommended to regularly examine the infection status of horses, either individually or at least by monitoring pooled samples, and to monitor the success of treatment [11, 13]. For the diagnostic examination of horse faeces, different coproscopic detection methods are available, with the recommended method dependent on the helminth species, the requirements of the owner and horse characteristics, such as age, pasture access and co-grazing animals [14–17]. Quantitative and semi-quantitative methods have proven to be particularly suitable for the examination of horse faeces [18, 19]. Simplifications and better application options for diagnostic procedures have the potential to increase the willingness of horse owners to use these procedures for routine monitoring, which can contribute to a reduction of selection for anthelmintic resistance [11, 18, 20, 21]. To date, post-treatment sample analysis is rarely used and, hence, treatment success is not determined [11, 13, 18, 22]. Due to the widespread anthelmintic resistance in cyathostomins and *Parascaris* spp. [5, 11, 19, 23–26], this can be expected to lead to continued egg shedding and contamination of the environment with eggs produced by resistant worms.

Widely used semi-quantitative methods to analyse egg shedding intensity include flotation alone or combined sedimentation/flotation [17, 27, 28]. Various modifications of the McMaster procedure, which includes flotation of eggs in a counting chamber, presumably remain the most widely used quantitative approaches [29–31]. Typically, the number of eggs counted in a single chamber needs to be multiplied by 100 (multiplication factor) to obtain the number of eggs per gram of faeces (epg). This multiplication factor is simply the reciprocal of the amount of faeces that is actually present in the counting field, i.e. 0.01 g faeces per single counting field on a McMaster slide. Alternative names for this factor are conversion factor and detection limit [31]. However, routinely used protocols are applied with multiplication factors of 25–50, which is achieved by counting two to four counting fields per sample [18, 32]. The FLOTAC [29, 33] and Mini-FLOTAC [34–37] procedures represent improvements of the McMaster approach, and both use mechanical separation of floated eggs from debris below by rotating the upper part of the device by 90°. This rotation step moves the upper part of the floated material to a counting chamber. The absence of debris below the optical plain in which the eggs are found improves visibility of the eggs and thus specificity and sensitivity [7, 18, 38]. The Mini-FLOTAC approach has the advantage that it does not require centrifugation of the device, which FLOTAC does, while the multiplication factor to calculate epgs from raw egg counts is 5 or 10 (2 or 1 Mini-FLOTAC counting chambers, respectively, per sample). This is intermediate between FLOTAC (multiplication factor of 1 or 2, 1 or 2 FLOTAC counting chambers, respectively) and the McMaster procedure (multiplication factor of 50 or 100 for counting 1 or 2 chambers, respectively, on the McMaster slide) [18, 34, 37].

A new quantitative method for the coproscopic quantification of nematode eggs uses the FECPAK^G2^ instrument (Techion, Mosgiel, New Zealand), which is an image-based diagnostic platform for worm egg quantification in faecal samples [39, 40]. The idea behind the FECPAK^G2^ procedure is that the animal owners themselves process the faecal samples independently using a standardised and simple flotation technique. The FECPAK^G2^ instrument is basically a special type of microscope equipped with an electronic camera [39]. The digital microscope picture is uploaded and the evaluation of the pictures to obtain quantitative data on the epg is routinely performed by a certified technician at Techion.

The aim of the present study was to use equine faecal samples to compare FECPAK^G2^ (multiplication factor of 45 epg) with the established Mini-FLOTAC method (multiplication factor of 5 epg) and a combined sedimentation-flotation approach as a semi-quantitative method in terms of: (i) frequency of samples detected positive; (ii) correlation between results for egg shedding intensity; and (iii) simplicity of use. Among the available quantitative methods, Mini-FLOTAC was chosen in this study as it represents a compromise between labour-intensive methods with very high sensitivity (e.g. FLOTAC multiplication factor of 1) and methods with presumably lower sensitivity (e.g. McMaster with multiplication factors > 25). To compare the practicability of the methods for routine diagnosis, total and hands-on times required to perform the analyses were compared between the methods. Among the semi-quantitative approaches, the combined sedimentation/flotation was chosen, initially to compare performance of Mini-FLOTAC and sedimentation/flotation for detection of eggs of anoplocephalid tapeworms.

## Methods

### Study design

**Fig. 1:**
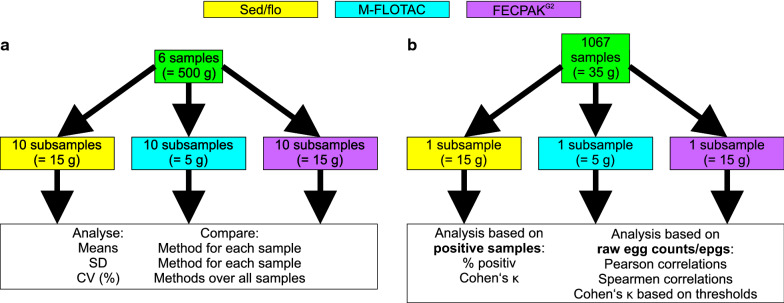
Graphic visualisation of the study design. In the first part of the study (**a**), precision of the three methods sedimentation/flotation (sed/flo), Mini-FLOTAC (M-FLOTAC) and FECPAK^G2^ was evaluated using six samples with varying numbers of epg. Each sample was analysed 10 times with each method. For each sample, means and SD values were compared for each method. Across all samples, CV (%) were compared between the methods. In the second part of the study (**b**), 1067 samples were all analysed once with each of the methods. Based on presence or absence of helminth eggs, the percentage of positive samples and inter-rater agreement, using Cohen’s κ values, were compared. Using quantitative data on epg or raw egg counts, Pearson and Spearmen correlations between methods were calculated and samples were assigned to categories of thresholds based on the quantitative methods Mini-FLOTAC and FECPAK^G2^. Assignment of samples to these categories was analysed using Cohen’s κ statistics. Abbreviations: CV, Coefficient of variation; epg, eggs per gram faeces; SD, standard deviation

Two separate sets of equine faecal samples were used in the present experiment. The first set of six samples was used to compare the precision of the three methods by examining each sample ten times with each of the three methods, i.e. sedimentation/flotation, Mini-FLOTAC and FECPAK^G2^ (Fig. 1a). In the second part of the study, a set of 1067 equine samples was analysed once with each of the three methods (Fig. 1b). Samples were analysed regarding the aspect of positivity for eggs of strongyles, *Parascaris* spp. and Anaplocephalidae using Cohen’s κ inter-rater agreement statistics. Quantitative egg count data were analysed on the level of raw egg counts (all three methods) and epgs (Mini-FLOTAC and FECPAK^G2^ only). Initially, mean epgs were compared, and Pearson and Spearman correlations were calculated for comparisons between methods for all three parasite groups. Since the number of samples positive for strongyle eggs was much higher than for *Parascaris* spp. and Anaplocephalidae, comparison of assignment of samples to certain epg categories by Mini-FLOTAC and FECPAK^G2^ was only performed for strongyle data. Strongyle-positive samples were categorised using typical epg thresholds (epgs of 50, 100 and 200), and inter-rater agreements and error rates for assigning a sample to different categories by the two methods were calculated (Fig. 1b). Finally, times required to obtain data from faecal samples for each method were compared.

### Horse faecal samples

In an initial experiment, the precision of all three methods was evaluated using six faecal samples. For the main data set, the faecal samples came from horses from private stables or herds as well as from clinics and veterinarians from all over Germany. Geographically, 75% of the samples came from the federal states of Brandenburg and Berlin, with 693 samples from Brandenburg and 105 samples from Berlin (Additional file 1: Table S1). Out of 1067 samples included in the main data set, 469 were specifically collected for the present study, 426 were sent in as routine diagnostic samples to the Institute for Parasitology and Tropical Veterinary Medicine, Freie Universität Berlin (FU Berlin), while 172 samples came from a study by Jürgenschellert et al. [41]. The latter samples were investigated a second time for the present study, and no data from the project by Jürgenschellert et al. [41] were used here to avoid bias introduced by different investigators. Only samples containing at least 35 g of faeces (enough to perform all three methods) were included. The samples were stored at 4 °C for a maximum of 10 days until examination. For all samples, examination with the different methods was performed on the same day. Samples were thoroughly mixed in the sample bag or a bucket by manual kneading to ensure homogenous distribution of eggs in the sample. Then the amount of faeces required for the different methods was determined using a scale.

### Combined sedimentation/flotation

Combined sedimentation/flotation [14] was performed as routinely used in the diagnostic service of the Institute for Parasitology and Tropical Veterinary Medicine (FU Berlin). To analyse samples with the sedimentation/flotation approach, 15 g faeces were suspended in 40 ml water. The suspension was filtered through a sieve (mesh size: 0.8 mm) and the filtrate was centrifuged in a 50-ml centrifuge tube at 400 *g* for 10 min. The supernatant was decanted, and the pellet first resuspended in 12 ml saturated sucrose solution (specific density: 1.26) and then transferred to a 15-ml centrifuge tube. After centrifugation at 200 *g* for 10 min, a wire hoop was used to transfer three drops of fluid from the surface to a glass slide. This material was then inspected under a microscope at ×100 magnification. Eggs were identified as strongyle, *Parascaris* spp., *Strongyloides westeri*, *Oxyuris equi* and tapeworm eggs and counted. For each egg type the results were categorised negative if no eggs were found, + when 1–10 eggs were present, ++ for 11–40 eggs, +++ for 41–200 eggs and ++++ for > 200 eggs. Except for the experiments for determination of precision, counting was stopped after 200 eggs had been identified.

#### FECPAK^G2^

A test package was provided by Techion Group Ltd, Aberystwyth, Wales, to carry out the FECPAK^G2^ procedure. In principle, the FECPAK^G2^ method can be used with sample weights of 10–115 g. To enable better comparability between the methods, it was decided to use 15 g of faeces for FECPAK^G2^, which was mixed with 60 ml water. The suspension was poured through a pre-filter (mesh size: 1 mm) and allowed to sediment for 30 min. The supernatant was decanted (leaving 15 ml in the sedimenter) before 80 ml of a saturated NaCl solution (specific density: 1.2) was added. This suspension was filtered through sieves with mesh sizes 600 and 425 µm, respectively. Then, 450 µl of the filtered suspension were pipetted into each of the openings of a FECPAK^G2^ cassette that was immediately loaded into the FECPAK^G2^ microscope/camera unit. Pictures were acquired and processed using the FECPAK^G2^ Lab Software Group Build Version 3.3.0.0. Subsequently, the parasite eggs on the images were manually differentiated, marked and counted using the markup function of the FECPAK Lab software. One counted egg corresponded to an epg of 45 according to the multiplication factor of the method.

### Mini-FLOTAC

A modification of the Mini-FLOTAC procedure was used which does not use the Fill-FLOTAC device. Instead, 5 g faeces were weighed on a scale, suspended in 45 ml saturated NaCl solution (specific density: 1.2) using a wooden spatula and filtered through a sieve (mesh size: 0.8 mm). After mixing the suspension by stirring with a spatula, both chambers of a Mini-FLOTAC device were immediately filled using a single use Pasteur pipette. After a flotation time of 10 min, the top part of the device was rotated by 90°, which transports floated eggs from the floating chamber to the counting chamber. Both counting chambers on a device were counted under a microscope using ×100 magnification. The epg was calculated by multiplying the number of observed eggs with the factor five [42].

### Precision of the methods

In order to determine the precision for each of the three methods, i.e. sedimentation/flotation, Mini-FLOTAC and FECPAK^G2^, these methods were consecutively conducted on subsamples of the same well-mixed samples. For this purpose, 500 g faeces was used to perform all three methods 10 times per sample. For sedimentation/flotation, all eggs were counted, and counting was not stopped after 200 eggs were detected.

### Statistical analyses

For analysis of precision, numbers of raw egg counts were initially compared for each sample using a one-way analysis of variance (ANOVA), followed by Tuckey’s multi-comparison test in GraphPad Prism 5.03 (GraphPad Software, San Diego, CA, USA). Bartlett’s test for differences in variances was conducted pairwise for all comparisons of methods within the same sample using the bartlett.test function in R 4.0.2 (R Foundation for Statistical Computing, Vienna, Austria). For comparison of methods over all samples, the coefficient of variation (CV) was calculated for each sample/method combination. The CVs for all three methods were then compared using paired data structure as implemented in the Friedman test, followed by Tuckey’s post hoc test in GraphPad Prism.

For comparison of methods using the large sample set, samples were classified as positive or negative for an egg type based on the different coproscopic methods. Confidence intervals for proportions of positive samples were calculated as Wilson score intervals using the OpenEpi web platform. Pairwise comparisons of proportions (2 × 2 tables) were conducted using mid-*P* exact tests with the tab2by2.test function in the R package epitools 0.5–10.1. After pairwise comparisons of results for all three methods,* P*-values were corrected for multiple testing using the p.adjust function applying the “Holm” method in R.

Cohen’s κ statistic was calculated to evaluate the inter-rater reliability between two diagnostics methods. For this purpose, the web portal http://vassarstats.net/kappa.html (last visited 15 Feb 2021) was used.

For comparison of samples based on egg quantities, Pearson correlations and regression, as well as Spearman correlations were calculated using GraphPad Prism. Comparison of slopes between linear regressions was performed using the implemented* F* test. GraphPad Prism was also used to compare egg numbers using the Student’s t-test or an one-way ANOVA followed by Bonferroni post hoc test.

## Results

### Comparison of precision between different coproscopic methods

**Fig. 2:**
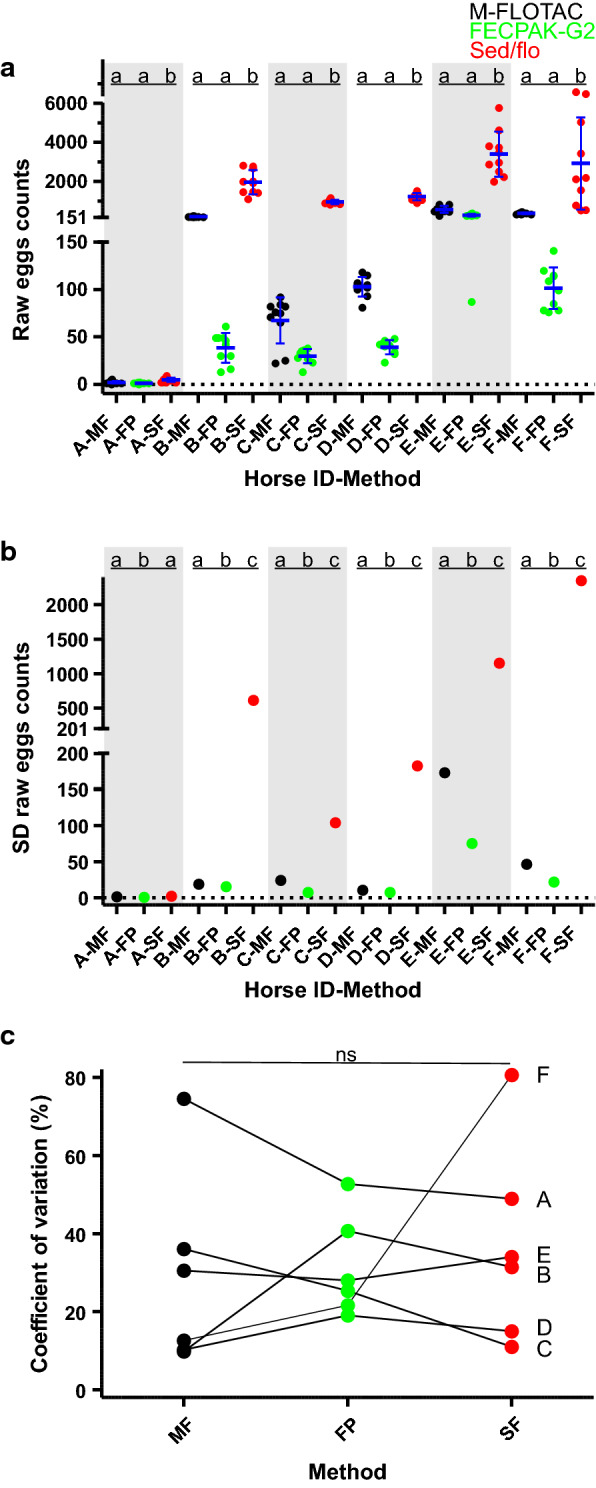
Comparison of precision between the Mini-FLOTAC (M-FLOTAC [MF]), FECPAK^G2^ (FP) and sedimentation/flotation (sed/flo [SF]) procedures. Faeces from six horses (A-F) were examined 10 times with each of the methods. **a** Raw egg counts of 10 replicates were compared for each horse separately using a one-way ANOVA followed by Tuckey’s post hoc test comparing all methods pairwise. **b** Comparison of SD for the 5 replicates was performed using Bartlett’s test. If data in **a** and **b** for the same horse and different method are labelled with the same letter (**a**, **b**, **c**), they are not significantly different (*P* ≥ 0.05). **c** CVs were calculated for each horse and method. No significant differences in CV were observed using a Friedman test for paired data. Abbreviation: ANOVA, Analysis of variance; ns, not significant

The results for raw egg counts for all three methods for six horse faecal samples analysed with each method 10 times are shown in Fig. 2a. Raw egg counts were significantly higher for the sedimentation/flotation procedure than for the other two methods for all six samples, as revealed by one-way ANOVA. For most samples, the variance was highest for sedimentation/flotation, followed by Mini-FLOTAC and FECPAK^G2^, with all differences between methods showing significant differences at *P* < 0.05, as determined with Bartlett’s test (Fig. 2b). The only exception was the sample with the lowest egg counts for which the variance for FECPAK^G2^ was significantly higher than that for Mini-FLOTAC and sedimentation/flotation while the difference between the latter two was not significant (Fig. 2b). CV values were calculated to compare absolute variation as dependent on the number of eggs counted. Values were in the range of 10–75% for Mini-FLOTAC, 19–53% for FECPAK^G2^ and 11–81% for sedimentation/flotation (Fig. 2c). A Friedman test, using data pairing according to the sample (horse) identifier, did not show any significant differences regarding the size of the CV between the three methods.

### Numbers of positive samples and faecal egg counts for different parasite groups

For the large dataset of 1067 samples, the number of positive samples per method and parasite species/group is shown in Table 1. By far the highest number of positive samples was found for strongyle eggs with all three methods. The number of samples positive for eggs of *Parascaris* spp. and Anoplocephalidae was at least tenfold lower than that for strongyle eggs (Table 1). Only very few samples were positive for *Strongyloides westeri* and *O. equi*. With FECPAK^G2^, detection of Anoplocephalidae and *O. equi* eggs has not been evaluated by Techion. Indeed, eggs of these species were found only in pictures of three and one sample, respectively. Therefore, FECPAK^G2^ data for Anoplocephalidae and *O. equi* were not further analysed. Table 1 also shows the range of epgs that were obtained using Mini-FLOTAC and FECPAK^G2^.

**Table 1:** Numbers, relative frequencies of samples positive for the respective helminth species/group and eggs per gram faeces in horse faecal samples (*n* = 1067) analysed using the Mini-FLOTAC, FECPAK^G2^ and combined sedimentation/flotation methods

Test procedures	Strongylidae	*Parascaris* spp	*Strongyloides westeri*	Anoplocephalidae	*Oxyuris equi*
*Mini-FLOTAC*					
Number	436a	43a	3	28	5
Frequency (%)	40.9	4.0	0.3	2.6	0.5
95% CI	38.0–43.8	3.0–5.4	0.1–0.8	1.8–3.8	0.2–1.0
Mean epg	97.3	5.3	0.44	0.9	0.4
SD epg	254.6	50.1	13.9	8.1	9.5
Range epg	0–2590	0–905			
*FECPAK*^*G2*^					
Number	317b	20b	4	3	1
Frequency (%)	29.7	1.87	0.4	n.a	n.a^a^
95% CI	27.3–32.8	1.2–2.9	0.1–1.0	n.a	n.a
Mean epg	116.5	2.8	0.17	n.a	n.a
SD epg	387.4	29.2	2.75	n.a	n.a
Range epg	0–905	0–630			
*Sedimentation/ flotation*					
Number	496a	49a	6	29	6
Frequency (%)	46.5	4.6	0.6	2.7	0.6
95% CI	43.5–49.4	3.4–6.0	0.3–1.2	1.8–3.8	0.3–1.2

CI, Confidence interval; epg, (number of) eggs per gram faeces; SD, standard deviation

Values in the same column followed by different lowercase letters (a, b) are significantly different (*P* < 0.01) in proportions (mid-*P* exact tested with *P*-values corrected using the “Holm” method)

^a^n.a indicates not available. The FECPAK^G2^ device has never been evaluated for detection of Anoplocephalidae and Oxyuridae and, therefore, the few positive samples were not used to calculate frequencies and other statistics

For all parasite groups, the highest number of positive samples was detected using the sedimentation/flotation method, followed by Mini-FLOTAC and FECPAK^G2^ procesures (if available). Nevertheless, not all positive samples were detected by sedimentation/flotation, and there were a few samples negative according to this method but positive according to the Mini-FLOTAC and/or FECPAK^G2^. For strongyle eggs, the frequency of positive samples was significantly higher with the sedimentation/flotation method than with the Mini-FLOTAC and FECPAK^G2^ using pairwise mid-*P* exact tests and* P*-value adjustment for multiple testing (Table 1). The difference between Mini-FLOTAC and FECPAK^G2^ was also significant. In contrast, for *Parascaris* spp., the numbers of positive samples were only significantly lower for FECPAK^G2^ compared to sedimentation/flotation and Mini-FLOTAC, whereas the difference between Mini-FLOTAC and sedimentation/flotation was not significant. The data clearly show that the semi-quantitative sedimentation/flotation method detected more samples as positive than the Mini-FLOTAC method, followed by FECPAK^G2^. Comparison of positive samples for Anoplocephalidae revealed no significant difference between the sedimentation/flotation and Mini-FLOTAC procedures.

### Comparison of coproscopic methods using inter-rater reliability analyses

To calculate Cohen’s κ coefficients for the different methods, a gold standard must be defined against which the methods are compared. Since the identification of strongyle and *Parascaris* spp. eggs during microscopy is quite simple, a specificity of 100% was assumed for all methods and, therefore, differences in results were assumed to be due to different sensitivities. This assumption is of course only an approximation and ignores small numbers of positive samples caused by contaminated laboratory equipment. However, in our routine diagnostic analyses of faecal samples from lambs and calves raised helminth-free, the frequency of false-positive samples is far below 1% and can thus be neglected here. None of the methods was able to detect all samples as positive. Thus, as the gold standard for this analysis, the set theory union of the results for sedimentation/flotation, Mini-FLOTAC and FECPAK^G2^ was used; that is, a sample was considered positive in the gold standard if it was positive in at least one of the tests. This approach is identical to the one proposed by Levecke et al. [43] for comparison of faecal egg counts (FECs) determinations of human samples. The results are shown in Table 2. Here, the high percentage of false-negative tests is the first obvious issue. Even with the most sensitive method, which is sedimentation/flotation, 5.3% and 10.9% of strongyle- and *Parascaris* spp.-positive samples were diagnosed negative. For the least sensitive method, slightly more than one third of the samples were false negative. Despite this relatively high number of samples considered to be false negative, Cohen’s κ coefficients for all methods were quite high. For both egg types, sedimentation/flotation showed almost perfect agreement with the highest inter-rater reliability, with the gold standard with Cohen’s κ values of 0.95 and 0.94 for strongyles and *Parascaris* spp. eggs, respectively [44]. This was closely followed by Mini-FLOTAC, showing strong concordance with the gold standard, with Cohen’s κ values of 0.83 and 0.87 for strongyles and ascarids, respectively. For FECPAK^G2^, Cohen’s κ values of 0.61 for strongyles and 0.52 for *Parascaris* spp. correspond to moderate and weak concordance with the gold standard [44].

**Table 2:** Cohen’s κ coefficients for sedimentation/flotation, Mini-FLOTAC and FECPAK^G2^ compared to the gold standard in 1067 equine faecal samples

Method	No. positive gold standard^a^ (frequency)	No. positive	No. false negative	Percentage of positive samples detected false negative	Cohen’s κ
*Strongyle eggs*	524 (49.1%)				
Mini-FLOTAC		436	88	16.8	0.83
Sed/flo		496	28	5.3	0.95
FECPAK^G2^		317	207	39.5	0.61
*Parascaris spp. eggs *	55 (5.2%)				
Mini-FLOTAC		43	12	21.8	0.87
Sed/flo		49	6	10.9	0.94
FECPAK^G2^		20	35	63.6	0.52
*Anoplocephalidae eggs*	31 (2.9%)				
Mini-FLOTAC	31	28	3	9.7	0.97
Sed/flo		29	2	6.5	0.95

^a^The gold standard was defined by counting every sample as positive that was diagnosed positive in at least one sample

Sed/flo, Sedimentation/flotation method

### Comparison of the quantitative methods FECPAK^G2^ and Mini-FLOTAC regarding FECs

**Fig. 3:**
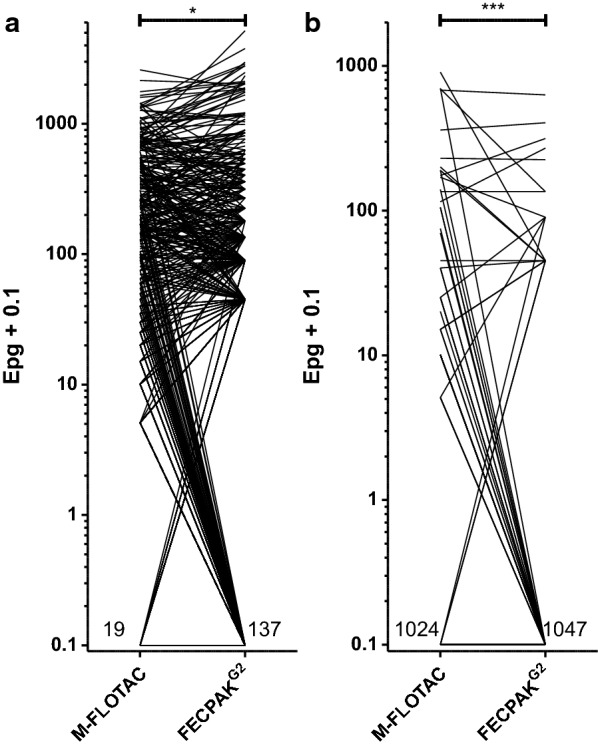
Comparison of number of epg obtained with the M-FLOTAC and FECPAK^G2^ methods for strongyle (**a**) and *Parascaris* spp. (**b**) nematode eggs. To allow a logarithmic presentation, 0.1 was added to all values. The number of samples negative for strongyle eggs was 19 for the Mini-FLOTAC and 137 for the FECPAK^G2^ method, respectively. For *Parascaris* spp., 1024 and 1047 samples were negative with the Mini-FLOTAC and FECPAK^G2^ methods, respectively. Even though more samples were positive with Mini-FLOTAC than with FECPAK^G2^, the mean number of strongyle epgs was significantly lower (*P* = 0.017) for Mini-FLOTAC than for FECPAK^G2^ applying a paired Student’s t-test. For *Parascaris* spp. epgs, higher mean epgs were measured with Mini-FLOTAC than with to FECPAK^G2^ (*P* = 0.0007). Asterisks indicate a significant difference at ***P < 0.001 and *P < 0.05

The epgs for strongyles and *Parascaris* spp. obtained from samples using Mini-FLOTAC and FECPAK^G2^ were initially compared using dot plots, as shown in Fig. 3. For strongyle eggs, a significantly lower mean epg was obtained using the Mini-FLOTAC method (97.1 epg) as compared to the FECPAK^G2^ method (116.5 epg) (Table 1; Fig. 3a). In contrast, the *Parascaris* spp. mean epg was significantly higher (*P* = 0.0007) with the Mini-FLOTAC approach (5.3 epg) than with the FECPAK^G2^ device (2.8 epg) (Table 1; Fig. 3b).

**Fig. 4:**
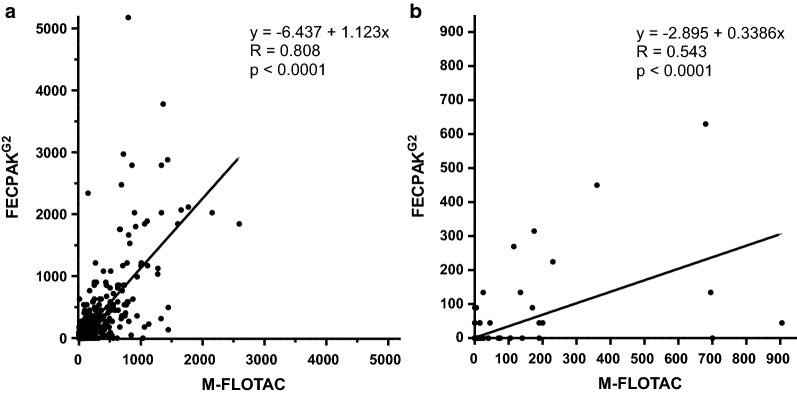
Pearson correlation between faecal egg counts obtained by the M-FLOTAC and FECPAK^G2^ procedures for strongyles (**a**) and *Parascaris* spp. (**b**). The linear regression function is shown as a continues line. The formula for the regression functions, the Pearson correlation coefficients and the* P*-values for showing a significant deviation of the slope from zero are shown in the graphs

In a second step, a Pearson correlation revealed a highly significant positive correlation for both parasite groups for data obtained by FECPAK^G2^ and Mini-FLOTAC, with a strong and moderate linear relationship for strongyles and *Parascaris* spp. epgs, respectively (Fig. 4). For strongyle epgs, the slope of the linear regression line was 1.123 (95% confidence interval [CI]: 1.062–1.185) and thus close to 1 (Fig. 4a). However, since a slope of 1 was not included in the 95% CI, this further corroborates that epgs for strongyle eggs were higher when FECPAK^G2^ was used compared to Mini-FLOTAC. In contrast, the slope for the linear regression was only 0.34 (95% CI: 0.31–0.37) for *Parascaris* spp. due to considerably lower epgs obtained using the FECPAK^G2^ method (Fig. 4b). In part, this effect can be explained by the relatively high number of samples that showed an epg of zero with FECPAK^G2^, while the samples were positive with Mini-FLOTAC.

### Comparison of the quantitative FECPAK^G2^ and Mini-FLOTAC procedures and with the semi-quantitative combined sedimentation/flotation method for strongyle egg numbers

In the sedimentation/flotation method, samples were assigned to one of five different categories (negative to ++++ [> 200 eggs]) based on the number of eggs counted under the microscope; however, counting was stopped once 200 eggs were found and the samples assigned to the highest category. Additional file 2: Fig. S1 shows a scatter plot with raw egg counts in the sedimentation/flotation methods on the* x*-axis and epgs obtained from Mini-FLOTAC and FECPAK^G2^ on the* y*-axis. These data show that very many samples fell into the ≥ 200 epg category and that parametric correlation analyses would be impossible for this data set. Therefore, two different approaches were used in the following analyses tests: (i) parametric correlation analysis including only samples with < 200 eggs counted in the sedimentation/flotation assay; and (ii) one-way ANOVA and Spearman correlation analyses using the epg categories in the sedimentation/flotation assay.

Using only the data set for which all eggs were counted (*n* = 951; excluding data with raw egg counts ≥ 200) in the sedimentation/flotation approach, Pearson correlation and linear regression analyses were performed with raw egg counts obtained from the Mini-FLOTAC and FECPAK^G2^ methods (Additional file 3: Fig. S2a). For this data set, a correlation of* r* = 0.699 between raw egg counts from the sedimentation/flotation and Mini-FLOTAC procedures was observed, which is just below the threshold of 0.7 for a strong correlation. For FECPAK^G2^, a moderate positive correlation (*r* = 0.574) with raw egg counts from the sedimentation/flotation method was found. The slope of both regression lines was significantly lower than 1 (calculated by comparison with a perfect correlation of sedimentation/flotation data with itself), which clearly indicates that the raw number of eggs counted with the sedimentation/flotation approach was higher than in both Mini-FLOTAC and FECPAK^G2^. In fact, the slopes of 0.392 and 0.037 directly indicate that the overall number of eggs counted in Mini-FLOTAC and FECPAK^G2^ was only 39.2% and 3.7% of the raw egg number counted in the sedimentation/flotation method. Furthermore, calculation of the ratio of the slopes for Mini-FLOTAC and FECPAK^G2^ versus sedimentation/flotation showed that the number of raw eggs counted with FECPAK^G2^ was only 9.5% of the raw number of eggs counted in Mini-FLOTAC. This is slightly lower than the 11.1% that would be expected from the ratio of the multiplication factors of 5 and 45 for Mini-FLOTAC and FECPAK^G2^, respectively. The same data are presented after calculation of strongyle epgs for Mini-FLOTAC and FECPAK^G2^ (Additional file 3: Fig. S2b). Of course, correlation coefficients and* y*-intercept remain identical whereas the slope of the curves increased by a factor of five and 45 for Mini-FLOTAC and FECPAK^G2^, respectively. As expected from the analyses of the raw data (Additional file 3: Fig. S2a), the slope for FECPAK^G2^ remained significantly lower (*P* = 0.006) compared to Mini-FLOTAC.

**Fig. 5:**
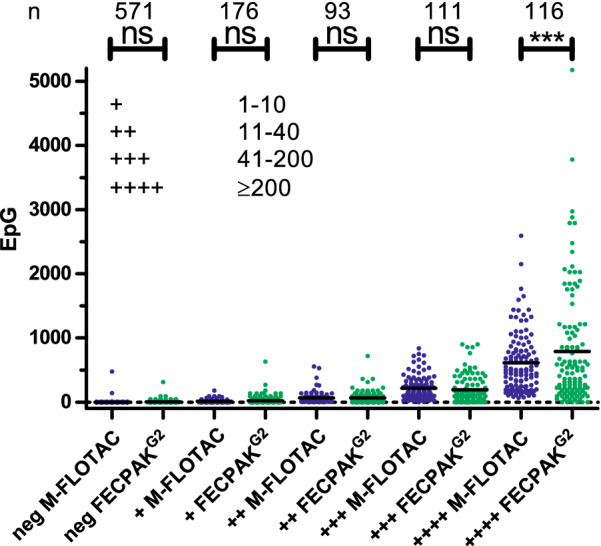
Comparison of strongyle faecal egg counts as epg for each category from the sedimentation/flotation method. Data were analysed by an ANOVA followed by Bonferroni post hoc test to compare M-FLOTAC and FECPAK^G2^ data within the same category from the sedimentation/flotation method. Means are indicated by black horizontal lines. Categories in the sedimentation/flotation method (negative to + [1–10 eggs], ++ [11–40 eggs], +++ [41–200 eggs], ++++ [> 200 eggs]) are assigned according to the number of eggs counted under the microscope. The number of samples (*n*) in each category is provided at the top of the figure. Asterisks indicate significance at ***P < 0.001; ns, not significant

In Fig. 5, strongyle epgs obtained by Mini-FLOTAC and FECPAK^G2^ are shown separately for each categorical result, including the highest category from sedimentation/flotation, which had been omitted in Additional file 3: Fig. S2a. Using an ANOVA, epgs were compared for all groups. This was followed by a Bonferroni post hoc test aimed at specifically comparing Mini-FLOTAC and FECPAK^G2^ data within the same category for sedimentation/flotation. Significant differences between Mini-FLOTAC and FECPAK^G2^ were only observed within the highest egg count class in the sedimentation/flotation method. In this group, the mean epg obtained using FECPAK^G2^ was 789.8 (range: 0–5175), which was significantly higher (*P* < 0.001) than that observed for Mini-FLOTAC (mean: 613.9; range: 65–2590). Despite the higher mean epgs, there were six samples with an epg of zero in the FECPAK^G2^ data from this group. In contrast, the lowest epg observed in the Mini-FLOTAC data was 65.

Spearman correlation was used to check for a significant correlation between the ordinal results from sedimentation/flotation and the epgs determined in the quantitative methods Mini-FLOTAC and FECPAK^G2^ (Table 3). A strong correlation was observed between the strongyle epg data obtained by the sedimentation/flotation and Mini-FLOTAC methods. The correlation between the respective data obtained by sedimentation/flotation and FECPAK^G2^ as well as between the two quantitative methods, Mini-FLOTAC and FECPAK^G2^, was only moderate.

**Table 3:** Spearman correlation comparing ordinal results for strongyle epg data obtained by the sedimentation/flotation method with numeric results for the Mini-FLOTAC and FECPAK^G2^ procedures

Strongyle eggs	Mini-FLOTAC	Sed/Flo	FECPAK^G2^
Mini-FLOTAC		< 0.0001^b^	< 0.0001^b^
Sed/Flo	0.914^a^		< 0.0001^b^
FECPAK^G2^	0.808^a^	0.770^a^	

^a^Spearman rank correlation coefficients ρ

^b^*P*-value for a monotonic correlation

### Differences between Mini-FLOTAC and FECPAK^G2^ regarding categorised outcomes

In terms of practical aspects, for targeted selective treatment it is often desired to categorise faecal samples according to a threshold epg, with the aim to identify horses that need treatment and those that do not [11, 18]. Since Mini-FLOTAC appeared to diagnose more samples as positive in the previous analyses in our study, this test was initially defined as the gold standard for the analyses in this section. Three different cut-off values were used: 50, 100 and 200 strongyle epg as determined by Mini-FLOTAC. For each cut-off, samples were categorised into groups below (e.g. < 50), the same or above (e.g. ≥ 50) the threshold according to the Mini-FLOTAC data. Then, FECPAK^G2^ strongyle epgs for each category were analysed (Table 4). These data show that, regardless of the chosen cut-off value, about 92–93% of the samples were placed into the same category by FECPAK^G2^ and Mini-FLOTAC (Table 4). Cohen’s κ coefficients were in the range of 0.707–0.777, corresponding to moderate agreement [44]. Thus, they are considerably higher than the Cohen’s κ values for agreement of FECPAK^G2^ with the gold standard, defined as ‘positive in one of the methods’, in terms of the question of whether samples were positive or not.

**Table 4:** Inter-rater agreement between data obtained using Mini-FLOTAC and FECPAK^G2^ to categorise strongyle egg shedding intensities

Category Mini-FLOTAC^a^	Category FECPAK^G2b^	Number^c^	% category	% Correct % Incorrect	Cohen’s κ	Mean^d^ Median^d^	Range^d^
< 50	Matching	777	72.82	92.03 7.97	0.777	3.2 0	0–45
	Higher	32	3.00			135.0 90	90–630
≥ 50	Lower	53	4.97			21.2 0	0–45
	Matching	205	19.21			205.9 135	90–5175
- Error rate I (%)^e^		53/258		20.5			
- Error rate II (%)^f^		60/215		27.9			
< 100	Matching	828	77.60	91.94 8.06	0.731	6.7 0	0–90
	Higher	33	3.09			205.9 135	135–630
≥ 100	Lower	53	4.97			50.1 45	0–90
	Matching	153	14.34			714.4 405	135–5175
- Error rate I (%)^e^		52/206		25.2			
- Error rate II (%)^f^		59/189		31.2			
< 200	Matching	898	84.16	93.53 6.47	0.707	14.8 0	0–180
	Higher	26	2.44			432.7 315	225–2340
≥ 200	Lower	43	4.03			94.2 90	0–180
	Matching	100	9.37			957.2 630	225–5175
- Error rate I (%)^e^		43/143		30.1			
- Error rate II (%)^f^		43/121		35.5			

^a^Mini-FLOTAC data were used to categorise samples using three different thresholds (50, 100 and 200 epg)

^b^Samples were counted depending on whether FECPAK^G2^ placed the sample in the same category (matching) or in another (higher, lower)

^c^Number of samples out of* N* = 1067 in total

^d^Mean, median and range of the epg determined by FECPAK^G2^ in the respective category

^e^Percentage of samples classified as above the threshold by Mini-FLOTAC but below the threshold by FECPAK^G2^

^f^Percentage of samples classified as above the threshold by FECPAK^G2^ but below the threshold by Mini-FLOTAC

Regarding the quantitative data, it is remarkable that even among the samples in the > 200 strongyle epg category according to Mini-FLOTAC, eight samples tested negative using FECPAK^G2^ (Table 4). Depending on the threshold, between 20.5% and 30.1% of the samples that were found to be above the threshold using Mini-FLOTAC were assigned to the category below the threshold by FECPAK^G2^ (error rate I in Table 4). On the other hand, among the samples with strongyle epgs < 200 in Mini-FLOTAC, FECPAK^G2^ reached an epg of 2340 in one sample. Among the samples assigned to a category above one of the thresholds using FECPAK^G2^, between 27.9% and 35% were sorted into the opposite category by Mini-FLOTAC (error rate II in Table 4).

### Comparison of quantitative FECPAK^G2^ and Mini-FLOTAC and with the semi-quantitative combined sedimentation/flotation method for ***Parascaris*** spp. egg numbers

**Fig. 6:**
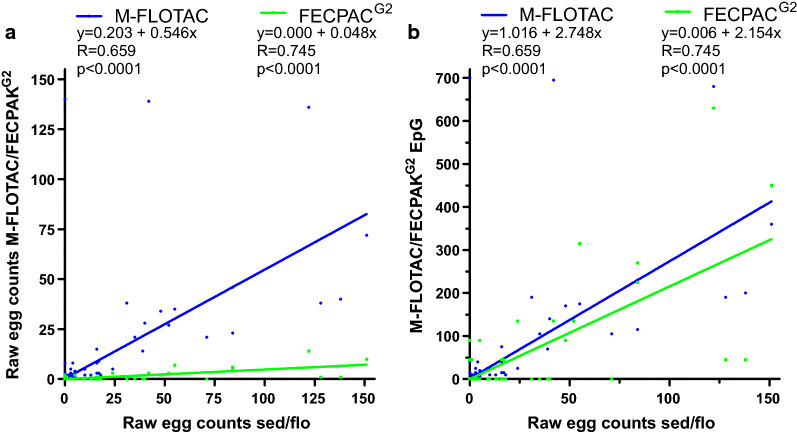
Pearson correlation between *Parascaris* spp. raw egg counts in the sedimentation/flotation procedure and results of the quantitative methods M-FLOTAC and FECPAK^G2^. Only data with raw egg counts in sedimentation/flotation ≤ 200 were included (*n* = 1066) since for a single sample with higher numbers no exact egg counts were available. Data from Mini-FLOTAC and FECPAK^G2^ are either shown as raw counts (**a**) or as epgs (**b**). Dots and regression curves for Mini-FLOTAC and FECPAK^G2^ are shown in blue and green, respectively. Linear regression equations, correlation coefficients and* P*-values (comparison to a slope of zero) are provided above the plots

In contrast to the data for strongyles, all except for one sample had *Parascaris* spp. raw egg counts in the sedimentation/flotation method of < 200. Since no exact raw egg count was available for this sample, it was excluded from the Pearson correlation/regressions. Pearson correlations were calculated between raw egg numbers for the sedimentation/flotation method versus either the Mini-FLOTAC or FECPAK^G2^ method (Fig. 6a). As for the strongyle data, correlations are also shown using epgs instead of raw egg counts for Mini-FLOTAC and FECPAK^G2^ on the ordinate (Fig. 6b). The slope of both lines was significantly < 1 as determined by comparison with a perfect correlation of sedimentation/flotation data with itself. The significantly lower slope for FECPAK^G2^ in comparison to Mini-FLOTAC (*P* < 0.0001) revealed that raw egg counts in Mini-FLOTAC and FECPAK^G2^ were only 54.6% and 4.8% of those obtained by sedimentation/flotation (Fig. 6a). Raw egg counts obtained by FECPAK^G2^ were only 8.8% of those in Mini-FLOTAC as determined from the ratio of the slopes. After calculating epgs from raw egg counts, the slope for the FECPAK^G2^ data remained significantly lower than for the Mini-FLOTAC data (*P* < 0.0001).

### Comparison of Mini-FLOTAC and combined sedimentation/flotation for egg numbers of Anoplocephalidae

Raw egg numbers for Anoplocephalidae were compared between Mini-FLOTAC and sedimentation/flotation (see Additional file 4: Fig. S3). Data show a strong correlation between raw egg counts obtained with both methods. The linear regression analysis revealed that the slope of the regression line was significantly lower than the slope of 1 of a perfect regression line of the sedimentation/flotation data with itself (*P* < 0.0001), showing that raw egg counts were significantly higher for the sedimentation/flotation method than for Mini-FLOTAC.

### Comparison of hands-on and total time required for the different methods

**Fig. 7:**
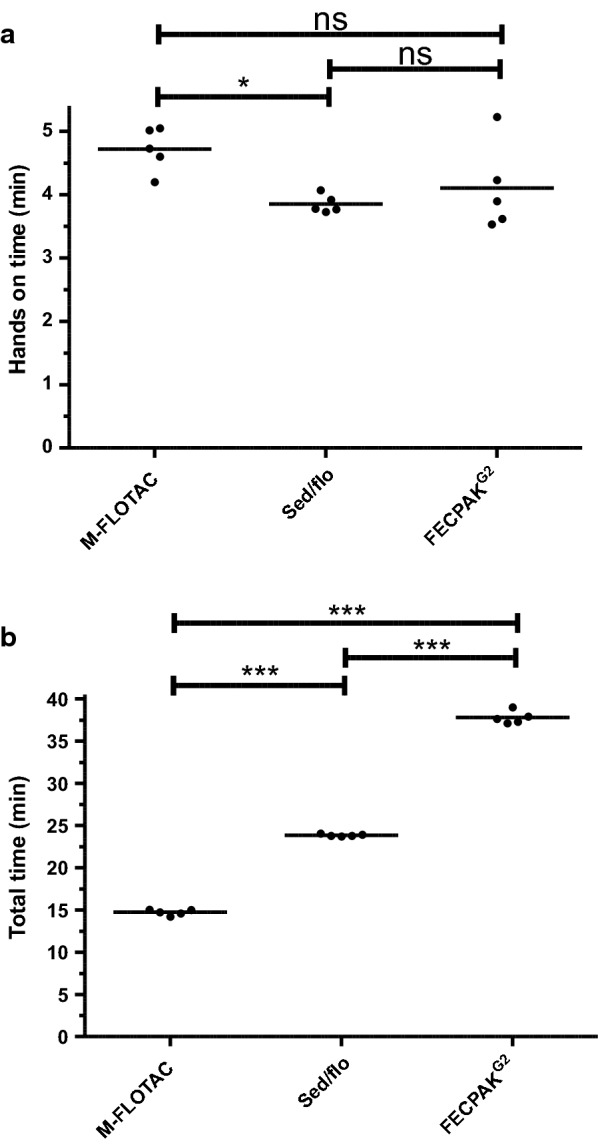
Comparison of hands-on (**a**) and total times (**b**) required for the M-FLOTAC, sed/flo and FECPAK^G2^ procedures. Individual times for each sample are indicated by dots and means are indicated by horizontal lines. Times were compared using one-way ANOVA followed by Tuckey’s post hoc tests comparing all combinations of methods. Asterisks indicate significant differences at *P < 0.05 and ***P < 0.001; ns, not significant

Hands-on and total time required to conduct an assay are also relevant factors influencing the practicability and uptake of the different methods. Since these times can easily be determined, five samples (with a strongyle epg range according to Mini-FLOTAC of 0–185) were examined with all three methods, and hands-on as well as total times required for the tests were measured. As shown in Fig. 7a, differences in hands-on times between the different methods were rather small. The only significant difference found was a slightly higher hands-on time for Mini-FLOTAC than for sedimentation/flotation. Total times required to process the samples with the different methods (Fig. 7b) included not only hands-on times but also the times required for any sedimentation and flotation steps, which was 10 min for flotation in the Mini-FLOTAC and 20 min (two 10-min centrifugation steps) for sedimentation/flotation. For FECPAK^G2^, a 30-min sedimentation step plus the time needed to acquire the image (approx. 3 min, 45 s) in the FECPAK^G2^ microscope were considered. Here, significant differences between all methods were found (Fig. 7b), with the longest total time required by FECPAK^G2^ (mean: 38 min) followed by sedimentation/flotation (mean: 24 min) and Mini-FLOTAC (mean: 15 min).

## Discussion

A wide range of different coproscopic tools is available to determine egg shedding intensity for gastrointestinal helminths. Flotation methods are widely used for many species but do not work for all parasitic helminths. For example, the Mini-FLOTAC method has only a very poor sensitivity for diagnosis of *O. equi* in comparison with the adhesive tape approach to detect eggs in the perianal region of horses [45], and this is probably true for all flotation methods. The principal coproscopic methods differ in terms of sample processing (e.g. direct evaluation of smears vs enrichment by flotation) and, thus, in potential losses of helminth eggs during processing, as well as the fraction of the sample that is actually counted (multiplication factor). Obviously, the methods also differ in the spectrum of parasites they can detect; for example the use of FECPAK^G2^ has so far only been described for the detection of eggs of parasitic nematodes, whereas Mini-FLOTAC is more multivalent and can also be used to detect eggs of cestodes and trematodes, protozoan cysts and oocysts and even some yeasts [36].

There have been considerable efforts in the last decade using both simulated and field epg data to establish statistical frameworks to estimate anthelmintic drug efficacies from FECs. The most advanced of these consider different hierarchical levels of variability, including: (i) biological variation between different individuals of a group; (ii) biological variation between different faecal samples or subsamples from the same individual; (iii) technical variation due to processing of the sample before counting; and (iv) random variation due to the assumed Poisson distribution of egg numbers in the counting device [46]. Levecke et al. [46] demonstrated that the precision for the epg estimate directly depends on the raw number of eggs that were counted. Similarly, the chance to diagnose a positive sample as false-negative decreases with: (i) increasing true epg; and (ii) decreasing multiplication factor to calculate epgs from raw egg counts [47]. The fact that raw egg counts were highest in sedimentation/flotation, followed by Mini-FLOTAC and FECPAK^G2^, in the data set where raw egg counts in sedimentation/flotation were < 200 is the simplest explanation for the sensitivity ranking. The sedimentation/flotation method uses the same amount of faecal sample as the FECPAK^G2^ method (15 g) while the amount is considerably lower for Mini-FLOTAC (5 g). This of course contributes to a higher sensitivity for the former. The second factor is the size of the subsample that is then actually investigated under the microscope. This number is unknown for sedimentation/flotation but is presumably much higher than for FECPAKG2 (2.22%). However, differences in losses during sieving, sedimentation and flotation might also contribute here, and in this context the different flotation solutions with different specific density used by both methods might also result in different raw egg counts. The difference in raw counts is also sufficient to explain a better correlation between the sedimentation/flotation and Mini-FLOTAC methods than between either of those methods and FECPAK^G2^. Moreover, there is a very simple and straightforward relationship between the magnitude of egg counts and the probability that a positive sample is tested negative. Hypothetically, if a sample has a true epg of 45 and there is no loss of eggs in any of the enrichment methods, the mean raw egg count using FECPAK^G2^ should be 1, whereas it should be 9 using Mini-FLOTAC. The observations of Levecke et al. [46] not only concern the mean number of raw egg count, but also the probability of whether at least one egg is identified. If Poisson distributions of observed egg counts with means of 1 and 9 are considered, then the probability of finding zero eggs (and, by extension, a false-negative diagnosis) is 36.8% and 0.012%, respectively. Thus, the multiplication factor can have a very strong effect on the chance to identify a sample as positive. Obviously, this effect is more pronounced for samples with low egg counts than for those with high egg counts. The fact that the mean epg for strongyle eggs was significantly higher for FECPAK^G2^ than for Mini-FLOTAC is no contradiction of this interpretation since this was only caused by high epgs in the  ++++ class.

The number of eggs recovered by a method, i.e. the raw egg counts, also depends on many additional factors, such as the amount of faeces used, the specific density of the flotation solution, the sieving system, any additional other factors influencing the loss of eggs during enrichment and the better “visibility” of eggs after enrichment compared to direct smears, such as Kato-Katz. While the methods compared in the present study all rely on flotation for enrichment, the flotation solutions differ. The sucrose solution with a similar specific density of 1.26 used for the sedimentation/flotation method might indeed contribute to the higher sensitivity of this method compared to the Mini-FLOTAC and FECPAK^G2^, both of which used a saturated sodium chloride solution (specific density: of 1.20) for flotation.

It is noteworthy that the two quantitative methods compared here differ greatly in terms of the multiplication factor used to calculate epgs from raw egg counts. With a factor of 5 for the Mini-FLOTAC and 45 for the FECPAK^G2^, it was to be expected that Mini-FLOTAC would outcompete FECPAK^G2^ in terms of sensitivity and precision. In comparison to the McMaster method, as the presumably most widely used quantitative method, where one slide is counted with two counting chambers per sample (multiplication factor 50), one could hypothesise that FECPAK^G2^ with a factor of 45 would perform similarly or be slightly more sensitive/precise. Rashid et al. [40] compared a modified McMaster method (multiplication factor of 15) with the same FECPAK^G2^ method used here regarding strongyle egg counts in alpaca (*Vicugna pacos*) samples. Using the same saturated sodium chloride flotation solution as used in the present study, the number of positive samples was significantly lower for FECPAK^G2^ than for McMaster while the mean epg was higher for FECPAK^G2^, although the latter value was not significantly different [40]. Applying FECPAK^G2^ (multiplication factor 45), Mini-FLOTAC (multiplication factor 10) and the McMaster method (multiplication factor 50) to human samples, Levecke et al. [43] showed that FECPAK^G2^ was inferior to Mini-FLOTAC and McMaster for strongyles (hookworms). However, it must be kept in mind that considerably larger amounts of faeces can be processed with FECPAK^G2^, which can be expected to increase its sensitivity.

The selective treatment for horses is the subject of controversial discussions [10, 11]**.** High levels of benzimidazole resistance in cyathostomins, the frequent occurrence of macrocyclic lactone resistance in *Parascaris* spp. and moderate frequencies of pyrantel resistance in both of these groups of parasites has led to the situation where no drug class can be used without the risk of experiencing a lack of efficacy. In Denmark [48, 49], Austria, the Netherlands, Germany and many other countries, prophylactic treatment with anthelmintics without a prior diagnosis of an infection with helminths has been prohibited, based on the argument that this policy will contribute to a decelerated selection of resistance [50, 51]. It has been advocated the treatment frequency should be reduced in order to slow down the selection of resistant worm populations [12, 52, 53]. If horses are only treated after a diagnosis of infection with a helminth, the importance of diagnoses will increase. However, epg cut-off values for treatment decisions are still being debated [54]. Such discussions address the issue of whether a general cut-off could be applied or if other aspects should be considered in the treatment decision including: (i) the age of the horses (foals and yearlings vs adult horses); (ii) the presence of *Parascaris* spp., which should not be assumed to be present together with strongyles due to its high pathogenicity in foals; (iii) the occurrence of highly pathogenic *Strongylus* spp. on a farm, which would discourage selective treatment; and (iv) the limited association between epg and the actual numbers of small strongyle worms in the gut [55]. Another important aspect regarding the usage of a threshold for treatment decisions is the question of reproducibility among different diagnostic methods. In this context, the data of the present study are highly relevant. Despite the overall lower performance of the FECPAK^G2^ method in comparison to the Mini-FLOTAC method, approximately 92% of the samples were assigned to the same category identified by the other method, regardless of whether an epg threshold of 50, 100 or 200 epg was used. Thus, despite sometimes astonishing differences in epgs, the treatment decisions based on epgs would not differ much between Mini-FLOTAC or FECPAK^G2^, regardless of the applied epg threshold.

Interestingly, the sedimentation/flotation method was found to have the highest raw egg counts and the highest Pearson correlation with the Mini-FLOTAC method, when based only on data with raw egg counts < 200 for the sedimentation/flotation method. Hence, one interpretation is that the semi-quantitative method might be even more sensitive and precise than the Mini-FLOTAC method. However, such a conclusion is clearly premature since it is based on data obtained by a single researcher (HB), who performed all of the the analyses. The finding may well be different if data from different researchers/technicians were to be included in the analysis, since higher variability in semi-quantitative methods is to be expected, particularly if samples are analysed by people with differing experience [56]. Standard operating procedures for semi-quantitative approaches are necessarily less precise than those for quantitative approaches. For example, it is impossible to truly standardise how samples are collected with a wire loop from the surface of a sample after flotation; this procedure will remain a human factor leading to variations.

In the present study, conclusions drawn from epg data for strongyles are more reliable than those for *Parascaris* spp., primarily due to the low number of samples positive for *Parascaris* spp. Nevertheless, the same ranking of methods in terms of number of positive samples was observed for *Parascaris* spp. as for strongyles. Considering the comparatively high pathogenicity of *Parascaris* spp. in foals, highly sensitive diagnostic tools should be recommended—particularly for stud farms with a known problem of parascariosis in foals [57, 58]. If quantitative results are needed on such farms, such as a FEC reduction test, more sensitive quantitative methods, such as FLOTAC (multiplication factor 1) would be an option to limit the effect of Poisson-distributed counting errors due to low raw egg counts.

One of the main reasons to choose sedimentation/flotation as the semi-quantitative method was the fact that it was reported to have a higher sensitivity than other coproscopic methods for the detection of eggs from Anoplocephalidae [59]. This egg type was only detectable using the sedimentation/flotation and Mini-FLOTAC methods; as yet, the FECPAK^G2^ approach has not been implemented for the detection of this egg type. Indeed, eggs were only rarely identified by FECPAK^G2^. Although there were no significant differences between both methods regarding sensitivity for Anoplocephalidae and inter-rater reliability when compared to the gold standard, raw eggs counts were significantly higher with the sedimentation/flotation method. This should result in significant differences in sensitivity due to the considerations discussed above for strongyle eggs. That significant differences in sensitivity between both methods were not observed in the present study might be due to the small number of samples positive for Anoplocephalidae [60].

Regarding the times required to conduct the analyses, there were no relevant differences between methods in terms of hands-on time. Although the Mini-FLOTAC procedure needed significantly longer hands-on times, this difference was less than 1 min. Thus, hands-on time is most likely not a relevant factor in the decision to use one or the other method or to calculate prices for diagnostic services based on the amount of work spent to obtain the results. For Mini-FLOTAC, hands-on times could be further reduced by using one of the Fill-FLOTAC devices, which were designed to collect a 2- or 5-g subsample that can be immediately homogenised in the device together with the flotation solution. In the present study, the time that could be saved using this device was not systematically determined. We used Fill-FLOTAC in previous studies, particularly when faeces or stool were analysed in field situations [61]. However, we decided against the use of Fill-FLOTAC in the present study since Fill-FLOTAC measures a volume and not a mass. For equine faeces, Boco et al. [62] reported that determination of the amount of faeces volumetrically with the Fill-FLOTAC led to an excellent agreement with the actual weight determined using a scale of 5.0 ± 0.11 g (range: 4.9–5.2 g), which corresponds to a maximal error of approximately 4%. This is not problematic as long as different samples are compared using the same method. In the present study, the same samples were analysed with different methods and, therefore, we decided to base all subsamples on weight. However, this also means that estimates of precision as provided here are presumably not applicable for the method using Fill-FLOTAC.

Whether the significant differences in total time required to conduct the investigations are of relevance depends a great deal on the organisation in the diagnostic laboratory. If the person who performs the analyses has the option to work on other samples/projects in parallel, this difference becomes of little importance (except for the obvious losses in efficacy when people need to work on multiple issues simultaneously). However, if the time required, such as the 30 min needed for sedimentation during FECPAK^G2^, which is the longest waiting time, is not used for other tasks, differences in overall times might be very important and contribute considerably to overall costs. For FECPAK^G2^, there is an additional waiting time between handling the samples and receiving the results of the picture analysis by Techion. Since this is not immediately cost relevant for the farmer or local veterinarian, it was excluded in the present study, but it must be clearly stated that fast turnaround of results would be highly advantageous for the final user and that FECPAK^G2^ has a disadvantage here in comparison to the other methods. However, if the time needed to send samples to a laboratory is considered, the uploading of images from FECPAC^G2^ and the electronic delivery of results might outcompete any parcel service handling faecal samples.

The decision of the horse owner or veterinarian to choose one method from among the spectrum of available diagnostic methods currently available, as well as the decision to perform FEC diagnostics at all, depends on many different factors. Some of these factors are based on scientific reasons while others are more related to convenience or economics. For foals, which havea high risk of infection with *Parascaris* spp., the most sensitive method would be advisable. For example, in the case of a suspected *Parascaris* infection, it is less important if the chosen method is quantitative or only semi-quantitative, since treatment of all positive cases is advisable, even if only a single *Parascaris* spp. egg is found [11, 22, 57]. In contrast, for a faecal egg count reduction test (FECRT) to identify drug-resistant nematode populations, a sensitive and precise quantitative method is required, and the Mini-FLOTAC method has been repeatedly shown to perform very well in FECRT studies [63–65], including studies on horses [32, 66–68]. Reasons to consider when choosing a test based on convenience include access to the laboratories that offer tests and acceptable turnaround times for results. For manual counting methods, such as those presented here, the time required to process a sample is directly related to labour costs, and high raw egg counts can be expected to increase the time required for counting. Recently developed automated counting methods, such as the Kubic FLOTAC [69], Parasight [15, 38, 70] or Telenostic for cattle [71], as well as smartphone-based solutions are currently being investigated and developed [15, 72]. The advantages of automated counting processes are shorter hands-on times, reduced requirements of personnel skills and training, lower personnel costs, reduced material costs and a reduced need for spatial capacities [23, 73–79]. More importantly, however, subjective fluctuations by examiners are eliminated, making the results more comparable [23, 28, 73, 74, 79]. The aspect of whether the processing of samples is performed by the farmer or in a laboratory where specialised technicians are paid for this purpose also directly contributes to the overall costs of a diagnosis and might be an advantage of FECPAK^G2^. The epidemiological situation can also influence the outcome of a decision process regarding the best suited diagnostic method. If epgs are known to be high, less sensitive methods that require less time or generate lower costs might well be sufficient, whereas more sensitive methods might be required if quantitative data are needed in settings with low egg shedding, for example to determine the resistance status of parasite populations in adult hosts.

## Conclusions

In the present study, the semi-quantitative sedimentation/flotation method turned out to be more sensitive than the quantitative methods Mini-FLOTAC and FECPAK^G2^. The order of sensitivities of the methods clearly corresponded to the order of number of raw eggs counted and the same holds true for the Pearson correlation coefficients between the methods. Despite its lower sensitivity, FECPAK^G2^ was able to consistently identify > 90% of strongyle-positive samples above the arbitrary thresholds of epgs at 50, 100 and 200 epg based on all samples including negative ones. Direct comparison of error rates between Mini-FLOTAC and FECPAK^G2^ on positive samples resulted in rates of between 20% and 36%, depending on the methods and applied thresholds, and were therefore considerably higher. Although Mini-FLOTAC performed better than FECPAK^G2^ according to the current dataset, FECPAK^G2^ can nevertheless be considered as an informative tool for treatment decisions against strongyle and ascarid nematodes in horses— particularly in cases where parasitological expertise is not available on-site.

## Supplementary Information

**Additional file 1: Table S1.** Raw data of all samples included in the study.

**Additional file 2: Figure S1.** Comparison of strongyle raw egg counts between semi-quantitative and quantitative methods. The scatter plot shows the raw number of eggs counted in the semi-quantitative sedimentation/flotation (sed/flo) on the abscissa compared to raw egg counts obtained with the quantitative Mini-FLOTAC (blue) and FECPAK^G2^ (green) methods on the ordinate. In the sedimentation/flotation approach, counting was stopped once 200 eggs had been identified.

**Additional file 3: Figure S2.** Pearson correlation between raw strongyle egg counts in sedimentation/flotation and results from the quantitative methods Mini-FLOTAC (M-FLOTAC) and FECPAK^G2^. Only data with raw egg counts < 200 using sedimentation/flotation were included, since exact egg counts were not available for higher numbers using sedimentation/flotation. Data from Mini-FLOTAC and FECPAK^G2^ were either shown as raw counts (**a**) or as eggs per gram faeces (**b**). Dots and regression curves for Mini-FLOTAC and FECPAK^G2^ are shown in blue and green, respectively. Linear regression equations, correlation coefficients and p-values (comparison to a slope of zero) are provided above the plots.

**Additional file 4: Figure S3.** Pearson correlation between Anoplocephalidae raw egg counts obtained using sedimentation/flotation and Mini-FLOTAC (M-FLOTAC) methods. Linear regression equations, correlation coefficients and* P*-values (comparison to a zero slope) are provided above the plots.

## Data Availability

All data generated or analysed during this study are included in this article and in the manuscript and its supporting information.

## Abbreviations

epg: Egg per gram faeces
M-FLOTAC: Mini-FLOTAC
