# Supplementary material for: Comparison of FECPAKG2, a modified Mini-FLOTAC technique and combined sedimentation and flotation for the coproscopic examination of helminth eggs in horses
Source: Parasit Vectors. 2022 May 12;15:166. doi: 10.1186/s13071-022-05266-y (PMC9097362; doi:10.1186/s13071-022-05266-y)
Supplement: Supplementary file 2 — Additional file 2: Figure S1. Comparison of strongyle raw egg counts between semi-quantitative and quantitative methods. The scatter plot shows the raw number of eggs counted in the semi-quantitative sedimentation/flotation (sed/flo) on the abscissa compared to raw egg counts obtained with the quantitative Mini-FLOTAC (blue) and FECPAKG2 (green) methods on the ordinate. In the sedimentation/flotation approach, counting was stopped once 200 eggs had been identified. [file 13071_2022_5266_MOESM2_ESM.pdf]

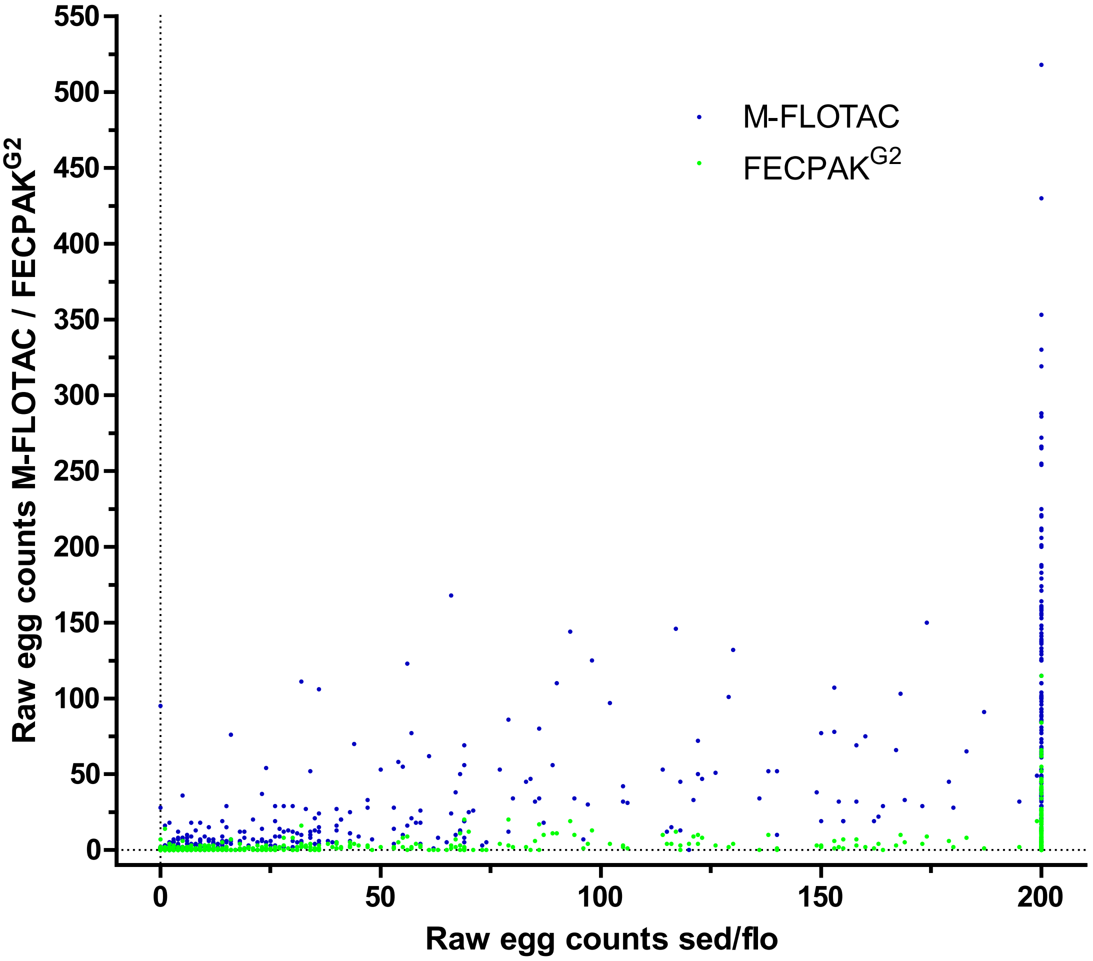

Additional file 2: Figure S1 Comparison of strongyle raw egg counts between semi-quantitative and quantitative methods. The scatter plot shows raw number of eggs counted in the semi-quantitative sedimentation/flotation (sed/flo) on the abscissa compared to raw egg counts obtained with the quantitative Mini-FLOTAC (M-FLOTAC, blue) and FECPAK^{G2} (green) methods on the ordinate. In the sedimentation/flotation approach, counting was stopped once 200 eggs had been identified.
