# Supplementary material for: Comparison of FECPAKG2, a modified Mini-FLOTAC technique and combined sedimentation and flotation for the coproscopic examination of helminth eggs in horses
Source: Parasit Vectors. 2022 May 12;15:166. doi: 10.1186/s13071-022-05266-y (PMC9097362; doi:10.1186/s13071-022-05266-y)
Supplement: Supplementary file 3 — Additional file 3: Figure S2. Pearson correlation between raw strongyle egg counts in sedimentation/flotation and results from the quantitative methods Mini-FLOTAC (M-FLOTAC) and FECPAKG2. Only data with raw egg counts < 200 using sedimentation/flotation were included, since exact egg counts were not available for higher numbers using sedimentation/flotation. Data from Mini-FLOTAC and FECPAKG2 were either shown as raw counts (a) or as eggs per gram faeces (b). Dots and regression curves for Mini-FLOTAC and FECPAKG2 are shown in blue and green, respectively. Linear regression equations, correlation coefficients and p-values (comparison to a slope of zero) are provided above the plots. [file 13071_2022_5266_MOESM3_ESM.pdf]

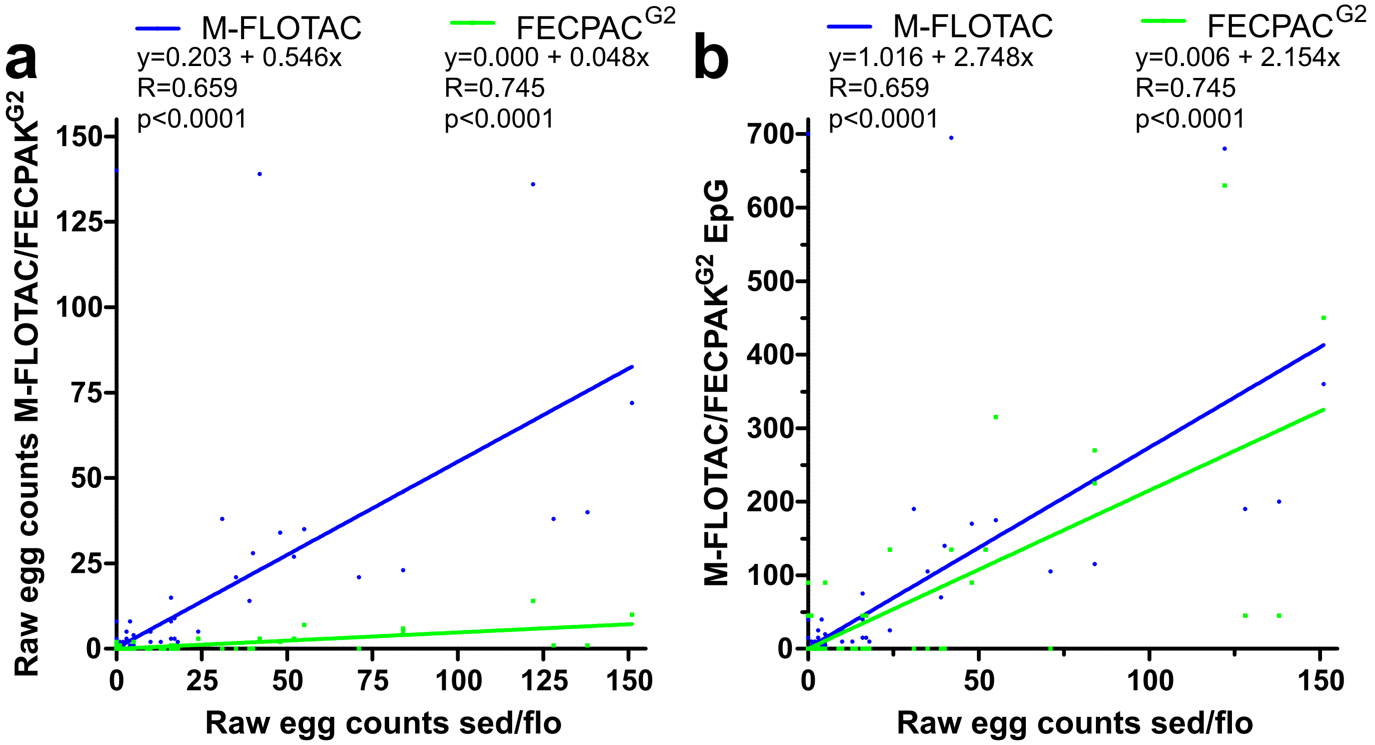

Additional File 3: Figure S2 Pearson correlation between raw strongyle egg counts in sedimentation/flotation and results of the quantitative methods Mini-FLOTAC (M-FLOTAC) and FECPAK^{G2}. Only data with raw egg counts in sedimentation/flotation < 200 were included since for higher numbers no exact egg counts were available for sedimentation/flotation. Data from Mini-FLOTAC and FECPAK^{G2} were either shown as raw counts (a) or as eggs per gram faeces (b). Dots and regression curves for Mini-FLOTAC and FECPAK^{G2} are shown in blue and green, respectively. Linear regression equations, correlation coefficients and p-values (comparison to a slope of zero) are provided above the plots.
