# Supplementary material for: Comparison of FECPAKG2, a modified Mini-FLOTAC technique and combined sedimentation and flotation for the coproscopic examination of helminth eggs in horses
Source: Parasit Vectors. 2022 May 12;15:166. doi: 10.1186/s13071-022-05266-y (PMC9097362; doi:10.1186/s13071-022-05266-y)
Supplement: Supplementary file 4 — Additional file 4: Figure S3. Pearson correlation between Anoplocephalidae raw egg counts obtained using sedimentation/flotation and Mini-FLOTAC (M-FLOTAC) methods. Linear regression equations, correlation coefficients and P-values (comparison to a zero slope) are provided above the plots. [file 13071_2022_5266_MOESM4_ESM.pdf]

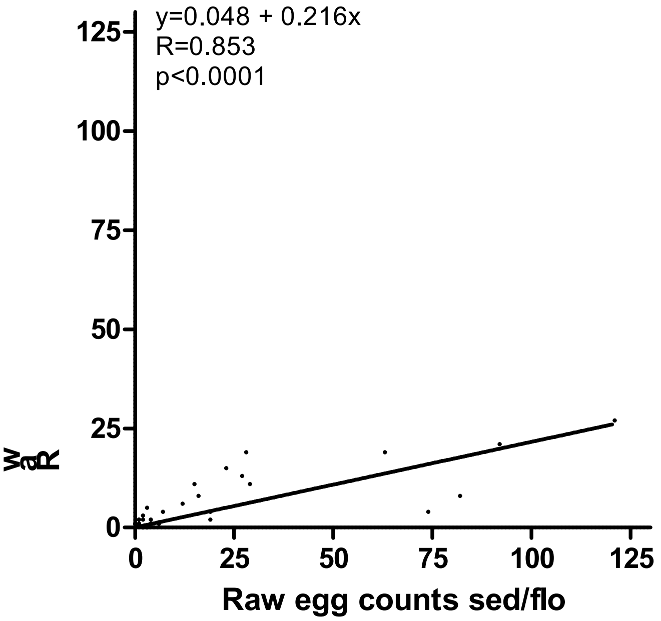

Additional file 4: Figure S3 Pearson correlation between Anoplocephalidae raw egg counts obtained using sedimentation/flotation and Mini-FLOTAC (M-FLOTAC) methods. Linear regression equations, correlation coefficients and p-values (comparison to a zero slope) are provided above the plots.
